# Exploring the Clinical Workflow in Pharmacogenomics Clinics: An Observational Study

**DOI:** 10.3390/jpm15040146

**Published:** 2025-04-05

**Authors:** Nicole Keuler, Jane McCartney, Renier Coetzee, Rustin Crutchley

**Affiliations:** 1 School of Pharmacy, University of the Western Cape, Cape Town 7535, South Africa; jmccartney@uwc.ac.za; 2 School of Public Health, University of the Western Cape, Cape Town 7535, South Africa; recoetzee@uwc.ac.za; 3 CHRISTUS St Michael Hospital, Texarkana, TX 75503, USA; russ.crutchley@christushealth.org

**Keywords:** pharmacogenomics, clinical workflow, pharmacist

## Abstract

**Background**: Pharmacogenomics (PGx) is the future of healthcare and implementation is being driven by increasing evidence. Understanding the workflow in a PGx clinic provides insight into the development and implementation of PGx services. It considers the patient’s perspective, the role of the interprofessional team and the pivotal input of the pharmacist. **Objectives**: The purpose of this study was to describe the clinical workflow followed in selected PGx clinics. **Methods**: Four different sites that offer PGx clinical services (United States of America) were included. Qualitative data were collected through semi-structured interviews and observations providing valuable insights into the workflow followed in both community-based and hospital-based PGx clinics. **Results:** Although each setting differed, the processes were similar with setting-specific workflows and barriers. This study highlights the role of the pharmacist and the interprofessional team, the resources used for interpretation of PGx test results and the importance of patient and healthcare education. **Conclusions**: Understanding the workflow and the role of the interprofessional team in PGx is essential to ensure successful implementation and sustainable precision medicine practices in resource-limited settings.

## 1. Introduction

Pharmacogenomics (PGx) is the future standard of care for individualized medication management [1,2]. Increasing evidence of the benefits of PGx supports clinical implementation [3,4,5,6,7]. However, successful implementation requires stakeholder engagement and is dependent on the setting, resources, health insurance and organizational structures [3].

Four main steps for PGx testing are recommended [8,9]: identify if the patient is a candidate for testing; order a test; interpretation and application of results to inform evidence-informed recommendations; and patient education.

Although clinical PGx implementation is spearheaded by pharmacists in the United States of America (USA) and Europe [10], collaboration with an interprofessional team is critical for successful implementation [9,11]. The core team should include a physician, pharmacist, nurse, genetic counsellor and laboratory specialists [12,13]. Healthcare providers play a key role in PGx implementation which includes ordering the test; interpreting results; communicating results to patients and the interprofessional team; and optimizing medication therapy [3]. Furthermore, healthcare providers play a critical role in community education and raising awareness.

Cicali et al. [9] describe a “one-visit” compared to a “two-visit” model for pharmacist-led PGx consultations based on experience at University of Florida Health and early adopters of PGx. With a “one-visit” model, the PGx test is ordered by a prescriber and the patient follows up with the pharmacist to conduct medication history, provide PGx education, discuss testing benefits and interpret results and implications. The pharmacist can also implement medication therapy changes, if accepted by the prescriber. In a “two-visit” model, the pharmacist first meets with the patient to conduct a thorough history, provides PGx education in terms of risk, benefits and limitations and determines the appropriateness of testing with recommended genes for testing. During a second visit, the pharmacist provides patient education, result interpretation and medication use implications. Phenoconversion [14] is an important concept that should be discussed and a patient-friendly handout should be provided. A “one-visit” model may be associated with increased efficiency and sustainability [15] and could be more feasible in resource-limited settings, such as South Africa. However, pharmacist-led consultations in collaboration with a physician improve patient care and result in higher patient satisfaction [16].

Although PGx implementation has been successful in developed countries, implementation remains limited in developing countries, like South Africa, due to unique challenges [16]. Pharmacists have been identified as important PGx role players in developed countries [11]; however, in South Africa, the pharmacist’s role in PGx has not been clearly defined and the implementation of clinical PGx services is in the very early stages.

The purpose of this study was to describe the clinical workflow followed in selected PGx clinics. Understanding the workflow provides insight into the development and implementation of PGx clinical practices and considers the patient’s perspective, the interprofessional team and the pivotal role of the pharmacist. This approach is valuable to gain understanding for implementation in resource-limited settings.

## 2. Materials and Methods

An observational design was used to map the current workflow in selected pharmacy-led PGx clinics. No similar PGx models exist yet in South Africa, and therefore the opportunity to observe current PGx practices was necessary.

Purposeful sampling, using professional networks, identified four different sites that offer PGx clinical services in Missouri, USA. The sites represented hospital and community pharmacy settings and were based on convenience and availability during the researcher’s limited time in Missouri. Site A is a research institute, linked to a specialized pediatric hospital. Site B is a PGx consultation business that offers pharmacist-led PGx consultations for direct-to-consumer (DTC) testing clients. Site C is a military hospital with a robust support system, and offers a structured PGx program with access to educational and financial resources. Site D is an independent community pharmacy.

Researcher-led observations of patient consultations and interprofessional team meetings were conducted (Site A), followed by semi-structured interviews with the pharmacist leading the PGx services at each site (Sites A, B, C and D) using a semi-structured interview schedule (Appendix A). During the interview, any clarifying questions relevant to the observations were asked. Sites B, C and D did not allow the researcher to conduct direct observations of PGx consultations due to privacy or limited opportunity, so data collection was limited to semi-structured interviews with the pharmacist. The direct observations were thus replaced with a stepwise description of the workflow in the clinic, provided by the interviewee. During the first interview, three additional questions were included in the interview schedule. An inductive approach allowed the generation of themes through observations and interviews [17].

**Figure 1:**
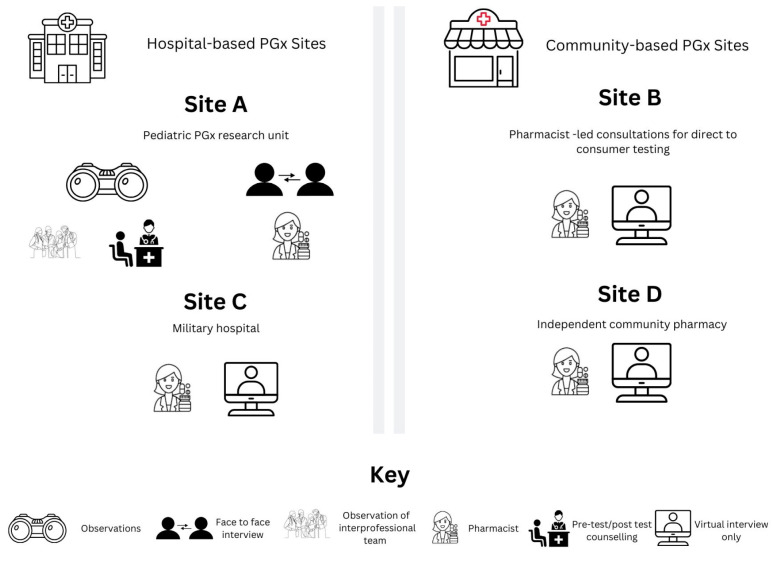
Summary of site-specific qualitative data collection processes.

The observations of patient consultations took place virtually since patient consultations were conducted via telehealth. Figure 1 below summarizes the site detail and data collection at each site.

Researcher-led interviews with the pharmacists were either conducted (i) virtually and recorded using Google Meet with pharmacists not in close proximity (*n* = 3), or (ii) face-to-face interviews (*n* = 1) which were recorded with a Samsung S24 smartphone. After the interviews were completed, the recording was transcribed using the Google Meet software or AI software on the Samsung S24 smartphone. The researcher listened to the recording and edited the transcript to ensure accuracy. An independent researcher then read the transcripts and coded in Atlas.ti. The researcher then re-read the transcripts and codes to identify additional codes and themes using thematic analysis. Thematic analysis encompasses identifying, analyzing, organizing and describing themes from collected data [18].

Site-specific observations and interviews informed the development of an algorithm representing the workflow at each site. The researcher shared the algorithm with the pharmacist via email for clarification and re-checking, after which the researcher made minor edits to reflect the approach followed.

The researcher utilized a reflection guide with standardized questions (adapted from Mack et al. [19]). The questions were answered by the researcher after each observation and interview as a debrief reflective exercise. The researcher also used a field book for documenting thoughts and ideas during observations and interviews. The development of the workflow was guided by a conceptual framework for process mapping [20] which guided the design and observations [21,22]. This approach helps to understand and represent the workflow of complex or novel systems.

## 3. Results

### 3.1. Demographics

All four pharmacists held a PharmD qualification and were employed as PGx consultants. Three pharmacists held academic positions: two as full-time lecturers at a university and one as a part-time lecturer at a continuing education institution. In the area of PGx, one pharmacist had a Master’s degree in Medical Genetics and was also a qualified genetic counsellor, while three pharmacists completed a certificate program in PGx, as described below.

*“Some certifications in PGx. University of Florida also had a one-year online graduate program and precision medicine”*

(P2, Community Pharmacist (CP))

*“They don’t yet have PGx certification through the board pharmacy specialties. …. there’s an organization that’s now called the American Association of Psychiatric pharmacists…. they had a training in Psych PGx. And then I did the ASHP PGx certificate program.”*

(P3, Hospital Pharmacist (HP))

*“Certificate PGx course through CE impact”*

(P4, CP)

Three pharmacists are members of the Clinical Pharmacogenomics Implementation Consortium (CPIC) [23]. One pharmacist is not a member of any PGx professional organizations, but attends regular PGx-focused continuing education opportunities.

The following four themes were identified during the analysis: (1) the role of the interprofessional team and the pharmacist; (2) patient and healthcare education; (3) resources used for PGx test interpretation; and (4) clinical workflow in selected PGx clinics.

### 3.2. The Role of the Interprofessional Team and the Pharmacist

The value of the interprofessional team and the pharmacist in PGx was observed in this study. In this study, the most common healthcare professionals were pharmacists and physicians (Sites A, C and D). The role of the interprofessional team is further highlighted in the clinical workflow and the Discussion Section. Discussions with the interprofessional team appeared patient-centred considering patient concerns and cost considerations.

Pharmacists fulfilled critical leadership roles at all sites. The roles and responsibilities of the pharmacists observed, included identifying patients who could benefit from PGx testing through medication reconciliation or referrals; pre-test counselling; history taking; interpretation of PGx results; sharing evidence-informed recommendations with the interprofessional team to optimize medication therapy; documenting and recording test results and recommendations in health records; post-test counselling; education of healthcare professionals and students; and implementing PGx services. Pharmacists can act as PGx consultants to patients, the healthcare team or industry. Considering all these roles, time management was identified as an important skill for pharmacists. The most important role for pharmacists as highlighted by Participant 4 was interpreting the result. All the pharmacists were involved in result interpretation, highlight the critical role of the pharmacist in this position.

*“Yes, the biggest thing would be the consultation. I am actually having to interpret the results. I get this whole panel and then I have to take that and determine what does that mean on prescribing. I am forming those whole recommendations.”*

(P4, CP)

PGx counselling requires extensive preparation, considering each patient is unique and recommendations are dependent on PGx results. Pharmacists can be consulted on creating the PGx report, especially for patients.

*“There’s the lab report, but then there’s report that they’re making that’s more personalized to the patient in the medications that they’re on… And then if the patients have any meds that they’re on where the genes are kind of in that red column, the higher risk, those patients are being flagged to recommend that they have genetic counselling, but everybody else is just getting a personalized summary report and so I’ve been helping with the generation of those reports.”*

(P2, CP)

Pharmacists can offer pharmacogenomic counselling to patients to make informed decisions about PGx testing. A pharmacist with knowledge in genetics could provide additional information to conduct informed counselling.

*“Being a genetic counsellor to me, it’s very important that people understand what the test can and cannot tell them and what some of the potential risks are or secondary findings of the testing.”*

(P2, CP)

Pharmacists created a personalized consultation note, highlighting results, recommendations and patient concerns, and generated a summary report for patients including drug–drug interactions.

*“So, what I do is on my consult report, it basically is a table that says the ‘here’s it’. Let’s say they were an ultra-rapid metabolizer (UM). I put there in UM and then I put what this means and some of the drugs that are affected. I try to tailor that to their current medications or conditions. I try to focus on why did the patient seek out this service in the first place.”*

(P4, CP)

### 3.3. Patient and Healthcare Education

Participant 3 highlighted the need for pharmacists to interpret the PGx results and to discuss the results with the patients.

*“Many times, when they (patients) have received them (PGx results). They said I looked at it, but I didn’t know what it means.”*

(P3, HP)

Patient education is critical; however, resources for patients were lacking during observations. Site C had site-specific educational and evidence-based resources as well as uniquely designed patient information resources.

*“We have a piece in education and supporting evidence-based medicine.”*

(P3, HP)

*“National reviewed guidance for different topics. So, we have one that’s sort of a general provider guide. We have some patient information sheets.”*

(P3, HP)

Participant 4 educated healthcare workers to assist with identifying patients who can benefit from testing. Therefore, education of healthcare professionals, including community healthcare workers, can assist the healthcare team in identifying patients.

One pharmacist has been invited to deliver talks on the importance of inclusion of secondary findings during patient counselling.

*“I’ve done a single lecture for the pharmacogenomics in the genetic counselling graduate programs. I’ve done a few for conferences, for the Missouri Pharmacy Association’s conference in August I’m doing an introductory to pharmacogenomics. That’ll be state-wide attended by pharmacists, pharmacy techs and pharmacy students.”*

(P2, CP)

Although pharmacists play a critical role, reimbursement is a challenge.

*“We can’t bill Insurance unless we’re doing it under some kind of collaborative agreement with a physician which I haven’t gotten to the point that I wanted to set such a thing up, so all cash pay so patients when they set up an appointment with me.”*

(P2, CP)

*“So, the biggest hurdles we’re facing are the billing.”*

(P4, CP)

For pharmacists working in community settings, billing for consultations is not possible due to law restrictions. Through a private consultancy/partnership, pharmacists can be remunerated.

### 3.4. Resources Used for Interpretation of PGx Test Results

The resources used to interpret PGx test results and inform evidence-based recommendations were explored.

*“We use PharmGKB, Pharmvar, I use a lot of Micromedex.”*

(P1, HP)

*“Not just the genes that have CPIC genes and then the other genes that are reported on and then I go to PharmGKB and the literature and trying to find anything else I can to help explain things for a patient.”*

(P2, CP)

Participant 4 also emphasized the importance of knowing how to use the resources.

*“You have to know how to utilize your resources. When I do the consultations, I reference a lot CPIC, PharmGKB.……I always want it to be evidence-based.”*

(P4, CP)

There are also resources to assist the healthcare team to make further informed decisions; Youscript was mentioned as an example.

*“So, it’s a software program that you can put the genetic information into along with all of the patient’s drugs and it will give you information about both drug and drug- gene interactions …. It’ll give you a list and you can quickly see which ones are green, yellow, red and sub them out quickly just to figure out which might be a good option.”*

(P2, CP)

**Figure 2:**
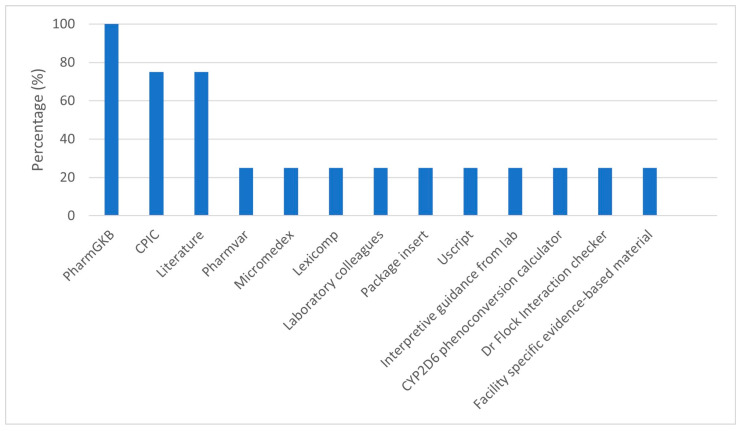
Evidence-based resources used by pharmacists (*n* = 4) working in PGx clinic.

Figure 2 below summarizes resources used in practice identified by the interviewed pharmacists. PharmGKB and CPIC were the most common resources consulted. One pharmacist mentioned consulting phenoconversion tools and one site had site-specific evidence-based material.

### 3.5. Clinical Workflow in Selected PGx Clinics

**Figure 3:**
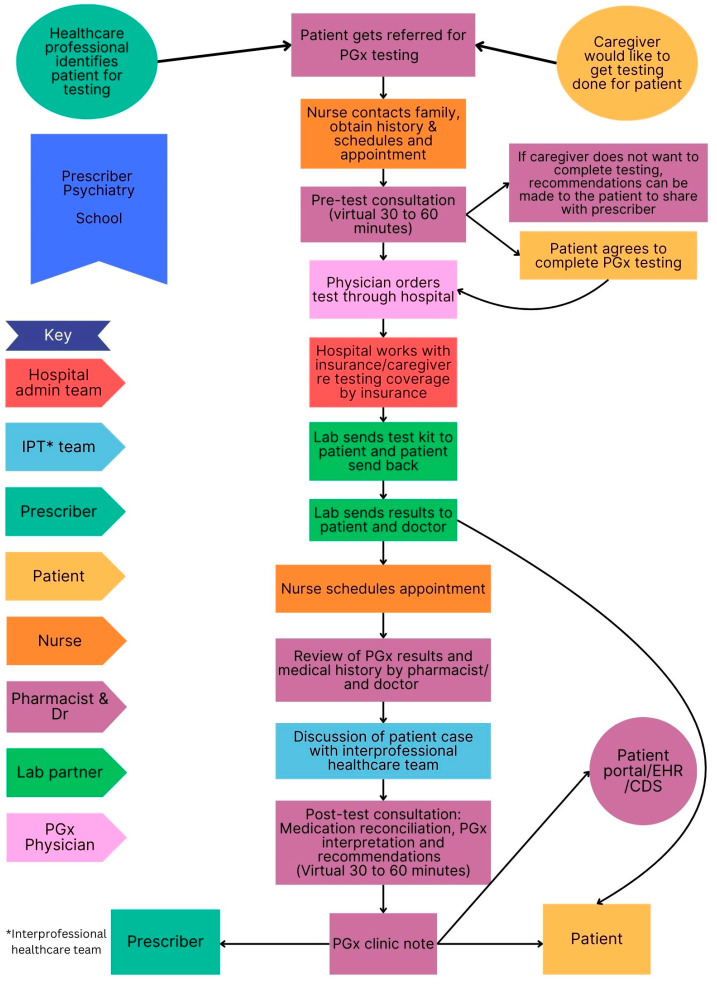
Flow diagram of workflow at a pediatric hospital (Site A). The interprofessional team consisted of a pharmacist, physician, nurse, specialists and laboratory personnel.

**Figure 4:**
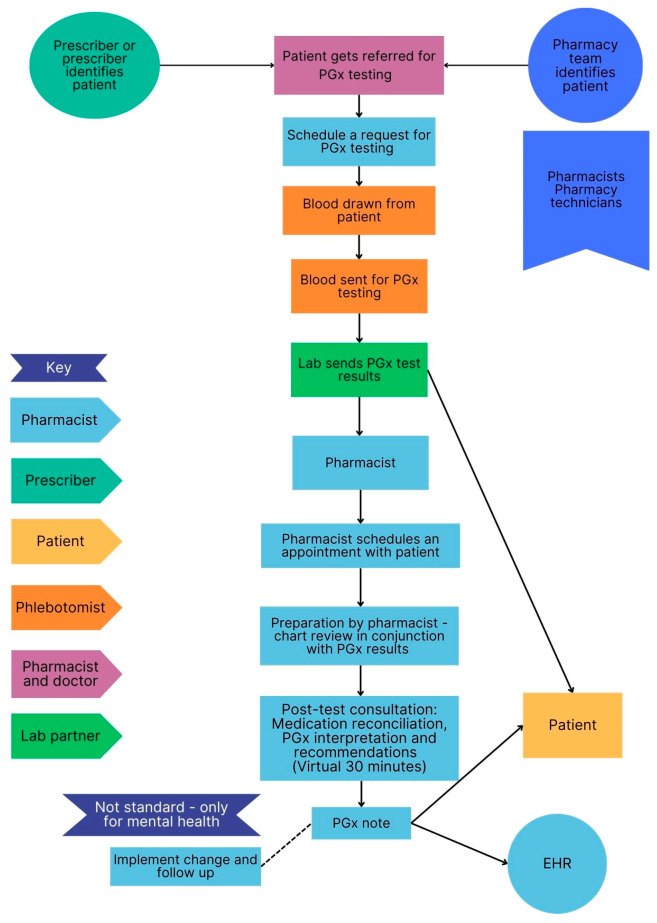
Flow diagram of workflow at a military hospital (Site C).

**Figure 5:**
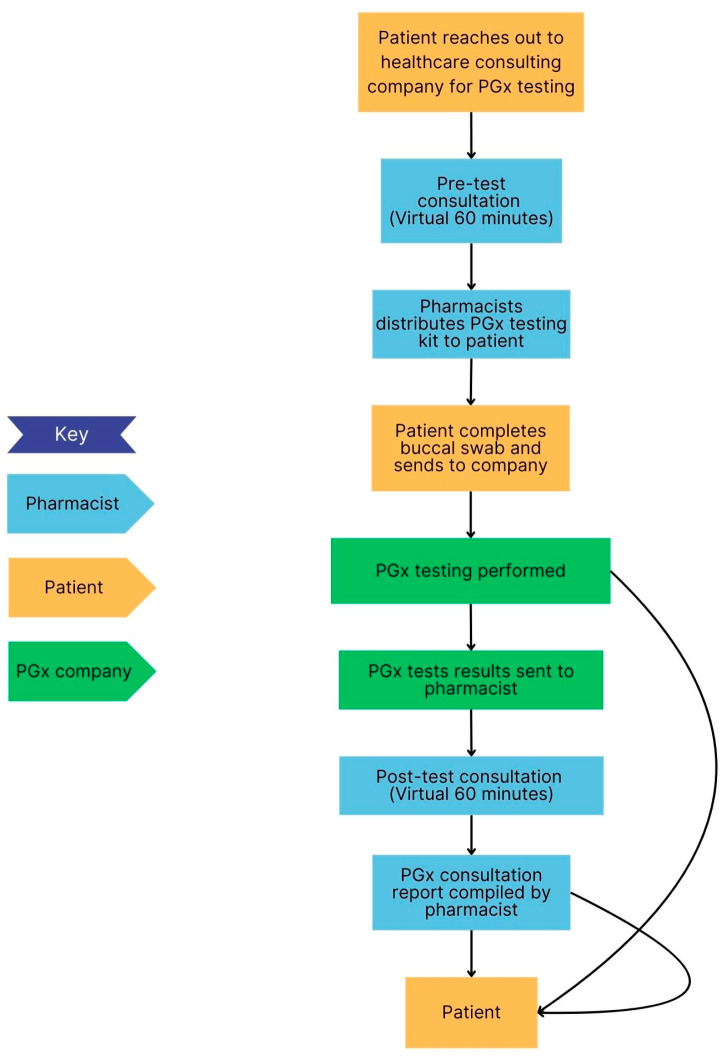
Flow diagram of workflow at direct-to-consumer testing (Site B).

**Figure 6:**
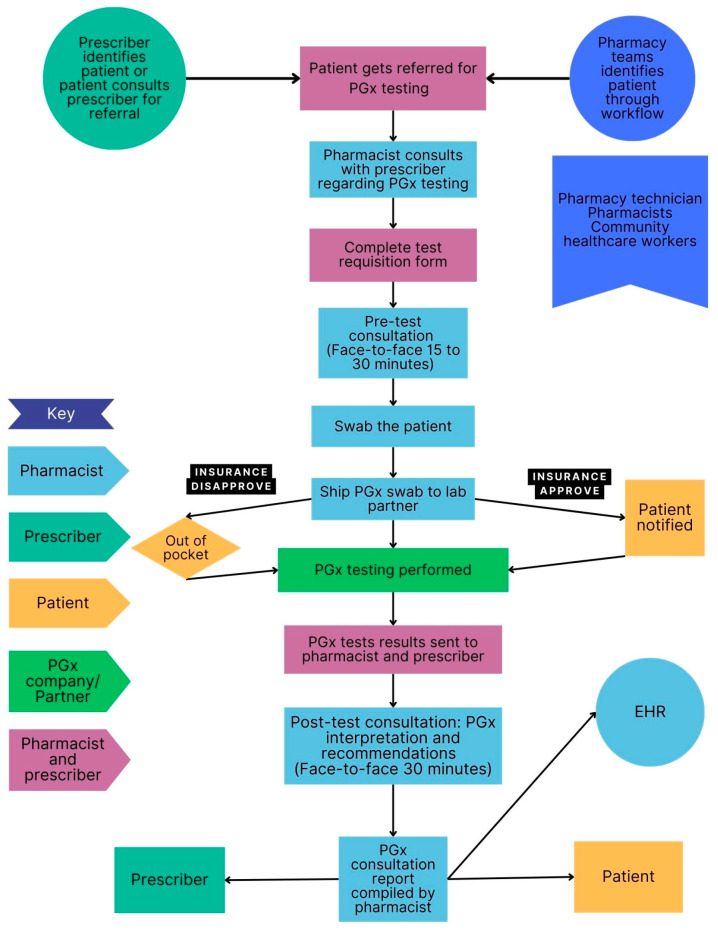
Flow diagram of PGx workflow at an independent community pharmacy (Site D).

The clinical workflow for each site is presented in Figure 3, Figure 4, Figure 5 and Figure 6 below. These sites are compared in terms of similarities and differences in Table 1 and PGx pre-test and post-test counselling is discussed in more detail in Table 2.

#### 3.5.1. Similarities and Difference Among Sites

Table 1 shows the similarities and differences between the settings and sites in terms of the type of counselling performed by pharmacists, PGx testing methods, payment options, the involvement of the healthcare team and distribution of the results. Pharmacists were always involved in post-test counselling and a buccal swab was the most common method used for PGx testing. Pharmacists were the most common healthcare professional involved in the patient’s PGx journey, while only one site had an interprofessional team involved in patient care. Most counselling was conducted virtually. The PGx results were always returned to the patient, however in a DTC model, it may not be possible to share the results with the prescriber or integrate the results into electronic healthcare records, which may limit the continuity of care. Payment options were similar across the settings.

#### 3.5.2. Pre-Test and Post-Test PGx Counselling

Three sites offered virtual PGx consultations, while one site offered face-to-face consultations. Pre-test and post-test PGx counselling varied at sites, approximately taking 30 min to an hour.

*“They’re slotted for 1 h……... It takes 30 min, sometimes it takes the hour.”*

(P1, HP)

*“I will normally set aside an hour for the pre-test consultation. Sometimes they don’t take that long. It just depends on how many questions the patient has and then for the post-test results I also usually set aside an hour, but those are more likely to take 30 min. I’ve had a few that take an hour.”*

(P2, CP)

*“It’s a 15-to-30-min conversation. And then after the results come back, it’s at least a 30-min conversation.”*

(P4, CP)

At Site C, healthcare providers are equipped to inform patients about PGx, which could eliminate the need for pre-test counselling by pharmacists.

*“Here, we tried to educate our providers to provide the consenting, I have left it open that if there was someone that had a lot of questions about testing, I’d be willing to meet with them initially”*

(P3, HP)

Table 2 summarizes the recommended discussion points for pre-test and post-test counselling informed by the interviews and observations. The direct quotations from interviewees are presented in italics with the interviewee’s unique identifier code and type of facility. Researcher’s observations and journal notes are presented in brackets.

The focus of the pre-test counselling is to discuss the reason for PGx testing and to make an informed decision. For the patient to make an informed decision, the reason for PGx testing is discussed as well as how the test can inform medication therapy. Taking medical and medication history is an important step to further support the motivation for PGx testing and to inform recommendations after PGx testing. Since PGx testing is not covered by all insurance companies, the method of payment is discussed. Lastly, patients need to provide consent for testing and to give healthcare professionals access to medical records.

The focus of the post-test counselling is to inform the patient about PGx results and how it influences current and future medication therapy. During the pre- and post-test counselling, pharmacists used PGx-related terminology to explain results and keep patients informed about the implications of PGx tests as well as the limitations.

## 4. Discussion

This study provides valuable insight into the workflow followed in PGx clinics. The processes remained similar at the sites with unique setting specific processes and barriers. Further, it highlights the role of the interprofessional team and the pharmacist in PGx clinics. The study findings provide valuable information and considerations for resource-limited countries which are planning PGx implementation.

A robust interprofessional team is key to successful implementation. Close collaboration between the physician and pharmacist was witnessed during observations at Site A, complementing each other with their individual knowledge and expertise. At the same site, the value of laboratory personnel in PGx test interpretation and validation were observed. Another valuable healthcare professional was the genetic counsellor observed at Site B. These observations emphasized the role of the interprofessional team in PGx services. Since interprofessional team meetings are educational opportunities, patient presentations should be structured to prevent confusion.

As highlighted by Participant 2, caution should be taken with the interpretation of results. The researcher noticed that PGx test reports were not presented in a standardized manner. Some, but not all of the test reports included the evidence that informs recommendations, for example, from resources like CPIC/PharmGKB/FDA. Some reports have a one-page patient PGx card summarizing the genetics results which can be helpful for continuity of care. During the observations, it was noted that certain genes could be categorized together; for example, cardiology/pain. The color coding of green, red and yellow for result interpretation was not always standardized and requires consideration when new research is conducted. These observations highlight the need for standardization of PGx reports to ensure accuracy and consistency, and that an evidence-based medicine approach is followed to interpret PGx findings. It is also important for PGx clinics to have a system in place for PGx results to be updated as more research becomes available. This is an opportunity for professional organizations to advocate for governance in PGx testing. To ensure clinical utility, only actionable gene–drug pairs should be tested.

Various PGx evidence-based medicine resources were used by pharmacists to inform recommendations. However, the FDA and Dutch Pharmacogenomics Working Group, highlighted as one of the expert guidelines to review when implementing PGx services [3], was not mentioned as a resource. Only one pharmacist mentioned phenoconversion tools, although considerations of drug–drug interactions were mentioned by other pharmacists. During the observations, the researcher appreciated that pharmacists were able to critically evaluate the literature to make evidence-based recommendations based on PGx results.

Kabbani et al. [3] state that it is the physician’s or pharmacist’s responsibility to return patient PGx results. Both the patient and healthcare professionals should report on the outcome of PGx-guided prescribing. Therefore, educating both healthcare professionals and patients is essential. Further, Cicali et al. [9] state that the patient is the owner of their data and has a right to have access to their data. At all the sites, patients were sent their PGx results with a personalized pharmacist report. These reports were shared with the provider, except for DTC testing; however, the patient could share it with the provider. Thus, communication with patients, including test interpretation and educational materials, is essential [24]. Pharmacists are encouraged to participate in the design and dissemination of implementation science and clinical PGx education, and to conduct PGx research [25]. Overall, it was observed that pharmacists followed a comprehensive approach to patient care with evidence-based recommendations, guided by a patient-centred approach.

In pharmacist-led PGx services, patient education is offered by pharmacists [9]. In this study, pharmacists were involved in educating patients, healthcare professionals and healthcare students. Further, they were responsible for result interpretation and provided recommendations to optimize therapy. During counselling, pharmacists provided an overview of PGx testing and its limitations. Options to cover PGx costs were discussed and history taking was always conducted to consider any changes based on PGx results. The importance of secondary findings and providing sufficient information to make informed decisions were also emphasized. Patient concerns were addressed as well as clinically relevant findings during counselling. A PGx consultation is an opportunity for pharmacists to provide broader counselling on other patient healthcare concerns. These observations highlight the pharmacist’s role in PGx clinics, especially during DTC testing, to provide counselling and interpretation which should be mandatory for PGx testing.

Although pharmacists provided personalized consultation reports, patient-focused educational resources were limited. Arwood et al. [24] acknowledged the lack of patient education and encouraged institutions to design material, if material is not available. Pharmacists can prepare patient-focused presentations for counselling sessions providing education in a visual manner which could assist with understanding PGx findings. Personalized consultation reports and patient education material can empower the patient and ensure continuity of care. Furthermore, the drug–gene interactions were mentioned, but not always discussed in detail. These are important discussion points, especially considering polypharmacy.

During the observations, it was highlighted that PGx is a tool to inform medication therapy across the patient’s continuum of care. To ensure continuity of care and to benefit from PGx testing, the results need to be integrated into the patients’ medical records and understood by healthcare professionals and patients. Electronic health records are important for continuity of care [3,9,26] and for PGx results to be considered in the future, for monitoring, evaluation and research purposes.

Although the pharmacists’ role was similar at all of the sites, some of their challenges were unique. In resource-limited settings, pharmacists could fulfil the observed roles with the necessary education and support. Klein et al. [12] identified common barriers for implementation: reimbursement; information technology; education; ethical, legal and social implications; and scientific considerations for implementation.

To ensure sustainability, it is recommended that pharmacists be reimbursed for their time when interpreting PGx results, communicating results with providers and patients and applying results to provide evidence-based informed recommendations [9]. Reimbursement was highlighted as a challenge in this study.

Other barriers to PGx implementation include country-specific barriers, which may include but are not limited to unclear regulatory frameworks of the gene–drug tests and the lack of consistency in reimbursement and healthcare policies between countries. Developing countries face various challenges for implementation, including clinical evidence, technology, policy and regulation, and human resources [27]. Since most of these consultations with patients were virtual, patients required access to data, electronic devices and knowledge to use these devices. In resource-limited settings, this method might be an obstacle to patient care. The most significant barrier to implementation is the education of healthcare professionals [12]. Most pharmacists in this study completed a certificate program and one had extensive on-the-job training. Globally, pharmacists should receive context-specific PGx education to fulfil their role and support implementation. However, limited educational opportunities exist in developing countries for pharmacists, which might further limit implementation.

Ethical, legal and social implications in PGx may be context-specific and should be outlined by governments and regulatory authorities. The World Health Organization outlines ethical, legal and social implications of PGx in developing countries [28]. Stratton et al. [29] highlight the importance of privacy, confidentiality, autonomy and informed consent in PGx testing. Comprehensive considerations for informed consent for PGx testing should be included in the pre-test counselling discussion. Healthcare providers involved in PGx testing should be aware of these ethical considerations. Returning results to patients in a patient-friendly manner [3] is a critical aspect of the ethical and legal implications of PGx testing and to ensure continuity of care.

Various approaches exist for clinical PGx implementation. A recent scoping review reports on 34 structured approaches [30] for PGx research and implementation. Most of the approaches were focused on the process to translate PGx research into practice and informed by stakeholders. Resource-limited countries should build on these approaches and collaborate with stakeholders who have experience to advance evidence-based PGx research and implementation locally [27]. Qualitative data collected in this study through observations and interviews with pharmacists leading PGx implementation allowed a unique experience to develop a global perspective for local implementation.

This study is not without limitations. Since only four PGx sites in the US were explored, the sample size is limited. Observations of PGx consultations with patients are valuable, but were only possible at one site. Developing countries face various barriers that have not been explored and therefore the presented workflow will have to be adapted to suit the need of the healthcare setting since the current observations were only performed in one area of the United States. Observations of other PGx clinics in developed countries should be considered.

## 5. Conclusions

Successful clinical PGx implementation programs are needed to advance personalized medicine to enhance patient safety, improve clinical outcomes, reduce healthcare costs, address educational needs and prepare for the future of precision medicine.

Understanding the workflow in PGx clinics and the role of the PGx interprofessional team is critical for sustainable precision medicine practices, especially in resource-limited countries.

## Figures and Tables

**Table 1:** Similarities and differences between sites and healthcare settings.

Description	Hospital		Community	
	Site A	Site C	Site B	Site D
Patient counselling performed by pharmacist				
Pre-test counselling	x		x	x
Post-test counselling	x	x	x	x
Virtual	x	x	x	x
Face-to-face				x
PGx testing method				
Saliva/buccal swab	x		x	x
Phlebotomy		x		
Payment method				
Out of pocket	x		x	x
Insurance	x		x	x
Other		x		
Involvement of healthcare team				
Pharmacist only			x	x
Pharmacist and physician				x
Interprofessional team (pharmacist, physician, nurse, specialists, laboratory personnel)	x	x		
Distribution of PGx results				
Electronic health records	x	x		x
Physician	x	x		x
Patient	x	x	x	x

The x indicates the relevance to the practice site.

**Table 2:** Pre-test and post-test PGx counselling.

Discussion Points	Description	Results from Interviews/Observations
Pre-test counselling		
Discuss reason for referral or need for PGx testing	Referrals may be from specialists, school, physician, patient self-referral, pharmacy technicians or community healthcare workers.	*“Yeah, so a lot of patients that have connected with me have done it via word- of-mouth. So other friends or family members have referred them to me. I’ve had a few people who have found me via Facebook or my website or LinkedIn. Trying to think if I’ve had any providers. No, but I haven’t marketed myself either locally to providers.”* (P2, CP) *“I’ve equipped them (pharmacy personnel) with different trainings and with these maybe keywords or phrases that might indicate that patients may need to visit with me regarding pharmacogenomics and some of those things include if they’ve had a bunch of changes in antidepressant medications; if they say something like I’m not gonna take that anymore. I’ve been having a lot of side effects or I don’t feel that this medication is working for me and that can be for various things. So, because pharmacogenomics is not one-size-fits-all, I can’t specifically say if the patient has this then they need pharmacogenomics. It’s very individualized. So, it’s more just by us listening and knowing our patients then one of our community health workers will get the patient in touch with me and all ask some more questions through that depending on what the patient may be having a concern with. So, let’s just say it is antidepressants that they don’t feel are working or they’ve been having side effects or whatever that looks like. I will then reach out to that prescriber of that medication or that class of medication so to initiate this request.”* (P4, CP) [Patient discussions appeared patient-centred)
History taking and medication reconciliation	Conducted through interaction with healthcare professional (pharmacist, nurse, patient/caregiver), and medical records.	*“I have kind of a detailed history that I asked for so I want information before I ever actually meet with them in terms of what are their current medicines, concerns any medicines that they’ve used in the past −do they have issues with them or did they not work? And just part of their health history as well.”* (P2, CP)
	Ask relevant questions related to symptoms, current medicines and concerns with past medication, including challenges and effectiveness.	*“And I always start with a medication reconciliation, very thorough one because I informed a patient that can influence how I interpret the results.”* (P3, HP) *“Our nurse when she calls the families to set up the appointment and she gets some history as well.”* (P1, HP) [At Site A, a nurse requests all the patient history for the physician and pharmacists and meets with the patient before a pre-test counselling session.]
	Family history (e.g., family history of cholesterol).	*“We can put the whole history together before appointment and then just ask questions to fill in the gaps where a provider’s notes might not have been very clear.”* (P1, HP)
Discussion with patient on what a PGx test is and available options so informed decisions can be made	A tool to optimize medicine and dosage. What the test can and cannot tell you and possibly secondary/incidental findings. Limitations of PGx tests.	*“Do a proper consent to the test because it’s a whole lot easier to talk to a patient about a problem. If you have discussed it up front then if you have never mentioned it and then you have to be, by the way, you have an APOE4 for variant that increases your risk of Alzheimer’s, something like that.”* (P2, CP) [During the patient counselling session, the researcher observed that the physician and pharmacist emphasized that PGx is only a tool to optimize patient outcome. Limitations of the test were also discussed; there might be insufficient evidence to support recommendations due to poor quality of research, e.g., trial design.]
Discuss the pharmacokinetic and pharmacodynamic process	Similar to explaining in post-test counselling.	[The physician and pharmacist communicated in a way that the patient understood. Discussion on pharmacokinetics and the role of drug metabolism. Discussed pharmacodynamics results and what they mean. Physician highlighted whether there is insufficient evidence to make an evidence-based recommendation. Lastly, drug–gene interaction table lists were shown. Discussion on, for example, which pain medication to avoid considering patient results, if applicable. Mentioned HLA genes that may cause allergic reaction.]
PGx terminology	Appropriate terminology should be used that is understandable for patients.	[During the researcher’s observation of patient consultations, the physician described the meaning of an intermediate, poor, normal and rapid metabolizer.]
Costs	Discussion on method of payment.	[PGx testing can be paid by insurance, by the patient or covered by the hospital due to service to the country (Site C only).]
Method of testing	Buccal cheek swab Phlebotomy	*“It is a buccal cheek swab… there’s certain instructions… I swab the patient on their cheeks.”* (P4, CP) [Patient can either receive the saliva test kit in the post or a pharmacist can perform a cheek swab.] *“Our lab does the phlebotomy.”* (P3, HP) [Only Site C uses phlebotomy for PGx testing.]
Ethical and legal implications	Patients need to give permission to conduct the testing, and provide healthcare professionals access to medical records	*“It has, of course, all the HIPAA information and protected health information of the patient”.* (P4, CP) *“I fill out as much as I legally am allowed to and then I send it over via fax, email− whatever the physician prefers and I send this information over asking for the diagnosis code in each supporting chart notes.”* (P4, CP) [Pharmacists work closely with physicians and the patient to access the necessary patient information while maintaining ethical and legal considerations.]
Post-test counselling		
Interpretation of test results	Discuss clinically actionable findings, drug–gene interactions, the implications and relevance to current medicines. Also highlight possible future implications and drug–drug interactions.	[The physician and pharmacist communicated in a way that the patient understood. Discussion on pharmacokinetics and the role of drug metabolism. Discussed pharmacodynamics results and what they mean. Physician highlighted whether there is insufficient evidence to make an evidence-based recommendation. The two actionable genes (CYP2C19 and CYP2D6) based on the patient’s current medication list were discussed. Lastly, drug–gene interaction table lists were shown. Discussion on, for example, which pain medication to avoid considering patients results, if applicable. Mentioned HLA genes that may cause allergic reaction.] *“How the results do and do not explain their concerns or questions.” (P2, CP)“Give them any recommendations that I might have to them to talk to their provider.”* (P2, HP)
Discussion of other non-PGx focused concerns	Discuss and address any other concerns the patient might have.	[How and when to take iron supplements. When to take the different formulations of methylphenidate.]
Knowledge re-check	Re-check the patient’s understanding of their results and implications.	[During observations of patient counselling sessions, the pharmacist and physician regularly asked the patient if they had any questions. Patients were overall very grateful for the information. ]
Limitations of results	Discuss limitations of findings.	[Laboratories reported on genes which do not have actionable recommendations; for example, some genes which are relevant to ADHD medication do not have actionable recommendations. These limitations were discussed with the patients.]

## Data Availability

Data collection tools and data are available upon request.

## Abbreviations

The following abbreviations are used in this manuscript:

CPIC	Clinical Pharmacogenomics Implementation Consortium
FDA	US Food and Drug Administration
PGx	Pharmacogenomics
PharmGKB	Pharmacogenomics Knowledge Base

## Appendix A

Exploring the clinical workflow in pharmacogenomics clinics: An observational study

**Semi-structured interview schedule**

We would like to know the step-by-step process followed in your PGx clinic, the services your facility provide, the steps that take places, who is involved in the different steps, roles and responsibilities.

1. To start, can you tell me your title and role here at (name of facility)
2. How long have you been in this role?
3. Could you please walk me though what happens when a patient visits your PGx clinic from first encounter to follow-up? We would like to know step-by step process and what takes place at each step, staff involved and the time it takes to complete the step.
4. What is your role in this process?
5. What qualifications do you have?
6. What skills do you require to fulfil your position?
7. What knowledge do you require to fulfil your position?
8. What does the first visit entail?
9. What is the typical duration of PGx consultations with patients?
10. Who (what staff roles) is involved in PGx testing and interpretation?
11. Who specifically performs patient counselling and medication reconciliation and at what point in the process? How does this happen—face to face or virtual?
12. How do you identify patients for PGx testing?
13. Do you offer any experiential learning opportunities for healthcare professionals or healthcare professional students at your facility?
14. Are you involved in any research in the field of PGx at your facility?
15. Are you part of any professional PGx organisations?
16. What resources do you use to assist with PGx result interpretation?

Additional probes to help with process mapping, if participant is highly involved in PGx testing, interpretation

Can you tell me how…?

What do you mean by…?

Process Mapping Observation Guide

During the interview/observation:

- Maintain the process perspective: What, where, how, roles involved in the process
- Define the scope of the process and identify any additional steps in the process
- Identify any additional decision points, loops, reworks, bottlenecks
- Identify process time at each step and between steps: best case scenario, worst case scenario, average time
- Identify if any movement takes place between the steps, such as walking to a different floor, carrying forms to a different office, etc.
- Define external and internal customers
- Identify any obstacles/barriers/wastes in the process
- Identify any variation in the process
- What are some replicable best practices/ facilitators to the current process?
